# Completeness and Quality of Neurology Referral Letters Generated by a Large Language Model for Standardized Scenarios

**DOI:** 10.3390/medicina61111931

**Published:** 2025-10-28

**Authors:** Watcharasarn Rattananan

**Affiliations:** Department of Family Medicine, Police General Hospital, 492/1 Rama I Rd., Pathum Wan, Bangkok 10330, Thailand; watrmd@gmail.com; Tel.: +66-(0)2-207-6217

**Keywords:** large language models, artificial intelligence, ChatGPT, referral letter, documentation, neurology, primary care, family medicine, consultation

## Abstract

*Background and Objectives*: Large language models (LLMs) offer promising applications in healthcare, including drafting referral letters. However, access to LLMs specifically designed for medical practice remains limited. While ChatGPT is widely available, its ability to generate comprehensive and clinically appropriate neurology referral letters remains uncertain. This study aimed to systematically evaluate the completeness and quality of neurology referral letters generated by ChatGPT for standardized clinical scenarios. *Materials and Methods*: Five standardized clinical scenarios representing common neurological complaints encountered in family medicine settings (headache, memory problems, stroke/TIA, tremor, radiculopathy) were used. Using a consistent prompt, ChatGPT (GPT-4o, 2025 release) generated 10 referral letters per scenario (50 letters in total). A dual board-certified neurologist and family physician scored the letters using a 30-point rubric across multiple domains: completeness (demographics, chief complaint, history of present illness, physical exam findings, management, and consultation questions) and quality (language level, structure, and letter length). Descriptive statistics and inferential analyses (ANOVA and Kruskal–Wallis tests) were applied to assess performance across scenarios. *Results*: The mean total score was 25.76/30 (95% CI: 24.85–26.67). Completeness averaged 87%, while language and structure consistently scored above 90%. Content gaps appeared in 36 out of 50 letters (72%), mainly in the history of present illness and physical examination sections. Variability was observed across letters, though not statistically significant between scenarios (ANOVA: *F* = 1.14, *p* = 0.352; Kruskal–Wallis: *H* = 3.52, *p* = 0.475). *Conclusions*: ChatGPT produced neurology referral letters of high linguistic quality but variable completeness, especially for clinically complex content. The variability pattern among letters reflected model inconsistency rather than case type. The reliance on a single rater and use of a non-validated rubric represent limitations. Future studies should include multiple raters, inter-rater reliability testing, and validated scoring frameworks. Ultimately, access to tailored LLMs exclusively trained for medical documentation could improve outcomes while safeguarding patient privacy.

## 1. Introduction

Advances in artificial intelligence (AI) have introduced transformative tools in healthcare, with earlier applications focusing on diagnosis, treatment, and prognosis [1,2]. Recently, large language models (LLMs) have emerged as potential aids in clinical documentation [3] or scribing [4,5]. Referral letters play a pivotal role in patient care coordination, bridging primary and specialty care. Accurate and complete referral letters are critical for effective communication between healthcare providers, particularly in neurology. For neurological patients, where complex patient presentations often necessitate detailed documentation, a well-crafted referral letter could significantly impact the patient outcomes. This group of patients seem to need more specialized medical LLMs tailored for neurological scenarios [6,7,8]. Unfortunately, access to medically specialized LLMs, especially for neurology, is not globally available.

ChatGPT, a prominent LLM, has been widely adopted across industries for text generation. Its potential for medical applications, including generating medical notes [9], discharge summaries [10], letter [11], and responses to patients’ questions [12]. Utilizing other LLMs in drafting physicians’ letters [13,14] has also sparked considerable interest. However, unlike tasks such as summarizing patient records or generating discharge instructions, referral letters demand a different structure, contextual understanding, and adherence to professional standards. Although LLMs have demonstrated proficiency in generating coherent text, their ability to address the needs of medical communication remains understudied. Most existing studies have focused on broader applications such as summarizing medical notes [15] or extracting information from electronic health records. This study narrows its scope to assess the specific task of generating referral letters, a critical component of interdisciplinary medical communication between primary physicians and neurologists. By examining the performance of ChatGPT in standardized neurological scenarios, we aimed to quantify the completeness and quality of AI-generated referral letters, identify common content gaps and areas for improvement as well as discuss implications for clinical practice.

## 2. Materials and Methods

### 2.1. Study Design

This study evaluated the ability of a large language model (LLM) to generate neurology referral letters from standardized clinical scenarios. Five scenarios were developed to mimic common neurological complaints typically encountered in family medicine practice:

Case 1: A 32-year-old female with chronic daily headaches, likely migraines, complicated by medication overuse.

Case 2: A 68-year-old female presenting with memory problems, concerning for early dementia.

Case 3: A 68-year-old male with transient ischemic attack (TIA) symptoms.

Case 4: A 68-year-old female with tremors suspected to have Parkinson’s disease.

Case 5: A 58-year-old male with right-sided radiculopathy consistent with L5–S1 involvement.

Each scenario was written in full sentences with relevant clinical details including demographics, presenting complaint, medical history, and examination findings. Emphasis on different angles of case information was applied depending on case types. For instance, a case with a memory problem provided relatively more detail in the history of present illness part, while a case with tremor or radiculopathy showed more physical exam findings. Combining the five cases, all parts of the analyzed medical information should be well-balanced. The full text of all five scenarios is provided in Appendix A to ensure transparency and reproducibility.

### 2.2. ChatGPT Letter Generation

Referral letters were generated using the free online version of ChatGPT-4o (OpenAI, San Francisco, California, USA; 2025 release) between February and March 2025. Each scenario was entered individually with the same prompt, and 10 letters per scenario were collected, resulting in 50 referral letters in total. The prompt was designed to resemble clinical situation in family medicine clinic where a specialized, medical LLM tool might not be available.

The exact prompt was as follows:

“I am a referring doctor, family physician. Please draft a neurology referral letter for the following patient, using standard medical referral letter format intended for a neurologist. The letter should include all necessary information about the case (e.g., history, physical findings, related laboratory or radiology data) and specify questions for consultation. The letter should be concise yet complete and written in a professional, clear tone appropriate for medical communication.”

Model parameters such as temperature, top-p, or stop sequences were not accessible through the online interface and therefore could not be modified. All outputs reflect the default system settings at the time of generation.

### 2.3. Evaluation Rubric

A 30-point rubric was designed to evaluate both completeness and quality of the referral letters (Table 1).

Completeness (20 points): demographics (2), chief complaint (2), history of present illness (5), physical examination findings (5), management prior to referral (3), and consultation questions (3).

Quality (10 points): language appropriateness (5), structural organization (3), and letter length adequacy (2).

The rubric was adapted from referral letter quality guidelines and refined with neurologist input to reflect essential elements of communication in neurology.

### 2.4. Scoring Procedure

Each letter was independently reviewed and scored by a physician dual board-certified in neurology and family medicine (the author). Scores for each domain were summed to produce a total score (maximum 30). *Language appropriateness* was determined using sum scores from ‘language level’ and ‘structure’, while *gaps in content* included scores from the demographics, chief complaint, HPI, and PE parts. In addition, qualitative observations of common content gaps and inconsistencies were recorded.

### 2.5. Statistical Analysis

Descriptive statistics (mean, standard deviation, and percentage completeness) were calculated for each rubric domain. Ninety-five percent confidence intervals (95% CIs) were reported for mean total scores.

Normality of score distributions was assessed using the Shapiro–Wilk test, and homogeneity of variance was evaluated using Levene’s test. Both completeness and total score distributions showed significant departures from normality (completeness: W(50) = 0.56, *p* < 0.001; total: W(50) = 0.64, *p* < 0.001). However, the homogeneity of variances was supported for both measures (completeness: F(4,45) = 2.12, *p* = 0.094; total: F(4,45) = 1.59, *p* = 0.194), indicating that group variances were not significantly different. Given these findings, both parametric (ANOVA) and non-parametric (Kruskal–Wallis) analyses were performed in parallel for robustness.

The overall effect sizes for group differences were moderate-to-large (Cohen’s f = 0.43 for completeness, 0.38 for total scores), corresponding to η^2^ values of 0.16 and 0.12, respectively. Because only overall scenario-level comparisons were performed, Bonferroni correction was not required. All tests used a two-tailed significance level of *p* < 0.05. All analyses were performed using manual computation confirmed with open-source statistical tools. Because the scoring process was conducted by a single evaluator, inter-rater reliability and intraclass correlation coefficients (ICCs) could not be determined, which is acknowledged as a methodological limitation of the present study

### 2.6. Ethical Considerations

No real patient data were used. All scenarios were synthetic and AI-generated, ensuring that no patient confidentiality was compromised. Institutional review board approval was not required.

## 3. Results

### 3.1. Overall Performance

Across the 50 referral letters, the mean total score was 25.76/30 (95% CI: 24.85–26.67; SD: 3.43). Scores ranged from 10 to 30, indicating that the LLM-generated letters can be vastly different (Appendix B). Demographic information and chief complaints were included in nearly all letters, with mean completeness rates of 96% and 95%, respectively.

Content gaps were present in 36 out of 50 letters (72%), most frequently in the history of present illness (HPI) and physical examination (PE) domains. Language quality and structural organization consistently scored highly, with mean ratings of 91.6% and 90%, respectively. Inferential testing demonstrated no significant difference in the total scores across the five scenarios (ANOVA: *F* = 1.14, *p* = 0.352; Kruskal–Wallis: *H* = 3.52, *p* = 0.475). This suggests that the observed variability was more related to the overall model output rather than case type.

### 3.2. Letter Completeness

Performance across the completeness domains showed variability (Table 2). Demographics and chief complaints were reliably included, with mean scores of 1.92/2 (96%) and 1.90/2 (95%), respectively. The history of present illness and physical examination findings were less consistently reported, with mean scores of 4.42/5 (88.4%) and 4.24/5 (84.8%), respectively. Omissions in these sections often involved absent negative findings. For instance, in the memory loss scenario, letters often omitted normal orientation function, while in other neurological cases, descriptions about negative family history of neurological disorders were inconsistently included.

The lowest-performing domain was management prior to referral, which achieved a mean score of 2.18/3 (72.7%). Documentation in this category varied widely, ranging from clear documentation of attempted therapies to minimal or absent descriptions of prior management. Case management discussions varied in depth, with some letters offering comprehensive assessments while others provided only minimal information. Often, the letters failed to provide detailed differential diagnoses or clear referral justifications. Some letters described a comprehensive assessment of alternative diagnoses (e.g., differentiating between TIA and migraine aura), while others failed to mention essential considerations.

Additionally, a notable inconsistency was observed in the *consultation questions*, which are critical for guiding specialist evaluations. Consultation questions scored an average of 2.8/3 (93%), though phrasing varied in clarity and specificity. While some letters provided direct and well-formulated referral questions (e.g., “Would DaTscan be beneficial in ruling out Parkinson’s disease?”), others lacked specificity.

Across the five scenarios, completeness scores showed no statistically significant differences (ANOVA: *F* = 1.24, *p* = 0.307; Kruskal–Wallis: *H* = 4.04, *p* = 0.401). This finding suggests that the observed variability was unlikely to be related to case type.

### 3.3. Letter Quality

The linguistic quality of the generated referral letters was consistently high across the five scenarios (Table 3). Language appropriateness scored an average of 4.58/5 (91.6%), reflecting fluency, grammatical accuracy, and a professional tone suitable for medical communication. Structural organization achieved a mean score of 2.70/3 (90.0%), indicating that most letters followed a logical format with clear sections for history, examination findings, and consultation questions. However, letter length was inconsistent, with 94% of letters either exceeding or falling short of the optimal word range (300–400 words).

While structural organization remained strong, some letters lacked a logical sequencing of information, particularly when presenting neurological examination findings or treatment plans. Furthermore, redundancies and extraneous wordings were observed in a subset of letters. Some letters repeated basic information without summarizing the assessment of the cases, reducing their clinical usefulness. In other cases, sequencing of the neurological examination findings lacked clarity, occasionally blending normal and abnormal findings without clear delineation. These issues, while minor, highlight the need for physician oversight to ensure that the letters maintain both precision and efficiency in real-world clinical use.

### 3.4. Summary of Findings

Overall, ChatGPT demonstrated strong performance in generating neurology referral letters across multiple standardized scenarios. The mean total score of 25.76/30 (95% CI: 24.85–26.67) reflected high overall quality, with strengths in demographics, chief complaint clarity, and language fluency. Completeness was somewhat variable, with frequent content gaps in the history of present illness and physical examination sections, affecting 72% of letters. The management domains were the most inconsistent, at times lacking sufficient detail to properly communicate to the specialist.

Inferential analyses confirmed that ChatGPT’s performance did not differ significantly across the five clinical scenarios. For total scores, the ANOVA results (F(4,45) = 1.14, *p* = 0.352) and Kruskal–Wallis results (*H* = 3.52, *p* = 0.475) both indicated non-significant differences between scenarios. For completeness scores, results were similarly non-significant (ANOVA: F(4,45) = 1.24, *p* = 0.307; Kruskal–Wallis: *H* = 4.04, *p* = 0.401).

Tests of assumptions supported these conclusions. While the Shapiro–Wilk tests indicated non-normal distributions (completeness: W(50) = 0.56, *p* < 0.001; total: W(50) = 0.64, *p* < 0.001), Levene’s tests confirmed variance homogeneity across groups (completeness: F(4,45) = 2.12, *p* = 0.094; total: F(4,45) = 1.59, *p* = 0.194). Given these patterns, the consistent results of both the ANOVA and Kruskal–Wallis tests indicate that observed score variability was not dependent on case type but reflected random model-level inconsistencies.

Taken together, these findings indicate that ChatGPT can reliably produce linguistically appropriate referral letters, though careful physician oversight remains essential to ensure completeness and clinical adequacy.

## 4. Discussion

This study systematically evaluated neurology referral letters generated by ChatGPT (GPT-4o, 2025 release) using five standardized scenarios common in family medicine. The results demonstrate that ChatGPT can produce referral letters that are consistently high in linguistic quality and structural organization. Essential elements such as demographics and chief complaint were reliably included, and language appropriateness exceeded 90% across scenarios. However, variability in completeness was evident, with 72% of letters containing content gaps, most often in the history of present illness and physical examination sections. Information about prior management was the least consistently reported domain. These deficiencies highlight that while the generated letters were readable and professionally written, they did not always contain the level of clinical detail expected in neurology referrals. Inferential analyses further showed that neither the total scores nor completeness scores differed significantly across case types, suggesting that the observed variability was not scenario-specific but instead reflected limitations of the LLM. While ChatGPT performed reliably across different neurological contexts, it did not fully adapt to the specific informational demands of the neurological cases. The variability in detail and content gaps could hinder care coordination, therefore, physician oversight is crucial.

Inferential analyses confirmed that ChatGPT’s overall performance was consistent across the five standardized neurological scenarios. Neither the total scores nor completeness sub-scores differed significantly among case types when tested with both parametric and non-parametric methods (total: ANOVA F(4,45) = 1.14, *p* = 0.352; Kruskal–Wallis *H* = 3.52, *p* = 0.475; completeness: ANOVA F(4,45) = 1.24, *p* = 0.307; Kruskal–Wallis *H* = 4.04, *p* = 0.401).

Tests of assumptions demonstrated that while the data were not normally distributed (Shapiro–Wilk *p* < 0.001 for both the completeness and total scores), the variances across groups were homogeneous (Levene’s test: *p* = 0.094 and *p* = 0.194, respectively). This justified the parallel use of ANOVA and Kruskal–Wallis analyses for robustness. Effect sizes were moderate-to-large (Cohen’s f = 0.43 for completeness, 0.38 for total), suggesting meaningful variability in absolute performance levels, even if scenario-specific differences were not statistically significant.

Taken together, these results indicate that ChatGPT’s variability was not dependent on case type but more likely reflected intrinsic model inconsistency. The observed non-normality is unsurprising given the limited dataset and the categorical nature of the scoring rubric, while the consistent findings across both statistical approaches strengthen the conclusion that ChatGPT performs with broadly similar quality across distinct neurological presentation types.

One of the study’s primary strengths is the use of standardized clinical scenarios, which allowed for controlled assessment and direct comparability across multiple AI-generated outputs. The evaluation framework, grounded in structured rubrics assessing completeness and quality, ensured that key aspects of referral communication were systematically analyzed. The inclusion of multiple cases covering common neurological presentations enhanced the study’s generalizability to various subspecialty referrals. Despite these advantages, certain limitations warrant consideration. While standardized scenarios facilitate objective assessment, they may not fully capture the complexity and variability of real-world clinical encounters, where patient narratives are often different and require adaptive reasoning. Additionally, the study focused exclusively on English-language outputs, limiting its applicability to multilingual healthcare settings where LLMs may exhibit a different performance based on the linguistic and cultural environment.

The study did not benchmark against human-authored documents or alternative LLMs. While this limits external interpretability, it is worth noting that the principal investigator is dual board-certified in neurology and family medicine, with extensive experience reviewing referral letters. This ensured clinical expertise and consistency. However, the use of a single evaluator introduces potential measurement bias; subjective interpretation cannot be completely excluded. The absence of independent, blinded raters, and inter-rater reliability analysis (e.g., Cohen’s kappa or intraclass correlation coefficients) limits the reproducibility of the findings. Future research should involve multiple raters and report the inter-rater reliability as well as intraclass correlation coefficients (ICCs).

The 30-point rubric, while grounded in clinical communication guidelines, has not yet been externally validated, which may limit its generalizability. Looking from a different perspective, this reflects a broader challenge in the field that is the absence of consensus frameworks for evaluating AI-generated medical communication. Other groups have suggested Delphi-style consensus methods or multi-specialist panels to establish reliability in scoring criteria, approaches that could be incorporated in future research. It is also important to mention that the reproducibility is constrained by using the free online version of ChatGPT-4o. System parameters such as temperature, top-p, or stop sequences were not accessible and may change over time as the platform is updated. Consequently, the exact outputs generated in this study may not be replicable in future sessions. Finally, statistical analyses were limited to descriptive summaries and group comparisons (ANOVA and Kruskal–Wallis tests). The sample size of 50 letters was modest, and although sufficient for initial exploratory analysis, larger datasets are needed to draw more precise conclusions regarding model variability and case-specific performance.

Future work should explore real-world integration. For instance, embedding LLMs into electronic health record (EHR) systems could facilitate the rapid generation of draft referral letters directly from structured patient data. Evaluations should also include workflow outcomes such as time savings, reduced administrative burden, and user satisfaction among physicians. Expanding the study to include multilingual scenarios, such as Thai, Arabic, Japanese, etc., would provide valuable insights into LLM performance across languages. Incorporating real-world clinical data and feedback from end-users would also promote understanding and development in the AI-assisted workflow. Since the performance of LLMs is dynamic and may change overtime [16], it is important to collaborate with AI developers to create affordable domain-specific models that can optimize outcomes while safeguarding patient privacy. Appropriate integration of such AI applications into EHRs is likely to reduce physician workload and burnout [17,18] as well as promote attitudes toward AI utilization [19].

Unfortunately, in developing or underdeveloped countries, the shortage of medical professionals and resources poses challenges to healthcare delivery. Inaccessibility to LLM applications specifically designed for medical practice is not uncommon. Language barrier can be an important obstacle in implementing LLMs in the medical service infrastructure of some countries. This is due to localized training data, and tailored algorithms are essential to ensure cultural and linguistic relevance. In these circumstances, careful use of free LLMs like ChatGPT or other open-source AIs may still be an option to alleviate documentation burdens and enhance medical communication.

## 5. Conclusions

This study demonstrated that ChatGPT (GPT-4o, 2025 release) can generate neurology referral letters of high linguistic quality and professional structure. Key clinical details such as demographics and chief complaint were reliably included, while gaps were common in the history of present illness, physical examination findings, and management prior to referral. The variability likely reflected intrinsic LLM limitations rather than case-specific challenges. These deficiencies highlight the need for physician oversight to ensure completeness and clinical adequacy. However, certain limitations of the study warrant careful result interpretation. Future efforts should prioritize real-world validation, seamless integration into clinical workflows, multilingual adaptations as well as accessibility to such AI applications in underdeveloped countries.

## Figures and Tables

**Table 1:** Rubric for evaluation of neurology referral letters.

Category	Description	Max Score
Demographics	Includes patient age, gender, handedness, and occupation	2
Chief complaint (CC)	Clearly states the main reason for medical visit	2
History of present illness (HPI)	Details about symptom onset, progression, triggers, impact, etc.	5
Physical examination findings (PE)	Findings from physical/neurological exams relevant to the case	5
Management (Mx)	Description of current or proposed management plan (investigation, treatment, referral, etc.)	3
Consultation questions (Q)	Specific questions for the specialist	3
Language level	Professional, clear, and concise language	5
Overall structure	Logical organization and separation of sections	3
Letter length	Adheres to the recommended word count range (300–400 words = 2; <250 or >450 words = 0)	2

Total maximum score: 30.

**Table 2:** Summary of completeness scores for each case.

Component	Case 1: Headache	Case 2: Memory Problems	Case 3: TIA	Case 4: Tremor	Case 5: Radiculopathy	Overall Score
Demographics (2 pts)	1.9 (95%)	1.9 (95%)	1.9 (95%)	1.9 (95%)	1.9 (95%)	1.92 (96%)
CC (2 pts)	1.9 (95%)	1.9 (95%)	1.9 (95%)	1.9 (95%)	1.9 (95%)	1.9 (95%)
HPI (5 pts)	4.3 (86%)	4.2 (84%)	4.3 (86%)	4.4 (88%)	4.5 (90%)	4.42 (88.4%)
PE (5 pts)	4.1 (82%)	4.0 (80%)	4.3 (86%)	4.5 (90%)	4.3 (86%)	4.24 (84.8%)
Mx (3 pts)	2.1 (70%)	2.0 (67%)	2.3 (77%)	2.4 (80%)	2.1 (70%)	2.18 (72.67%)
Q (3 pts)	2.8 (93%)	2.7 (90%)	2.8 (93%)	2.9 (97%)	2.8 (93%)	2.8 (93.33%)
Overall completeness score (20 pts)	17.4 (87%)	16.7 (83%)	17.8 (89%)	18.0 (90%)	17.5 (88%)	17.46 (87.3%)

**Table 3:** Quality metrics (language, structure, letter length).

Quality Metric	Case 1: Headache	Case 2: Memory Problems	Case 3: TIA	Case 4: Tremor	Case 5: Radiculopathy	Overall Score
Language level (5 pts)	4.6 (92%)	4.5 (90%)	4.7 (94%)	4.6 (92%)	4.5 (90%)	4.58 (91.6%)
Structure and organization (3 pts)	2.7 (90%)	2.6 (87%)	2.8 (93%)	2.9 (97%)	2.7 (90%)	2.7 (90%)
Letter length compliance (2 pts)	1.0 (50%)	1.1 (55%)	1.2 (60%)	1.0 (50%)	1.0 (50%)	1.02 (51%)
Total quality score (10 pts)	8.3 (83%)	8.2 (82%)	8.7 (87%)	8.5 (85%)	8.2 (82%)	8.38 (83.8%)

## Data Availability

The raw data supporting the conclusions of this article will be made available by the authors on request.

## List of Abbreviations

AI	Artificial intelligence
CC	Chief complaint
DaT	Dopamine transporter
EHR	Electronic health record
HPI	History of present illness
LLM	Large language model
Mx	Management
PE	Physical exam findings
Q	Consultation questions
SD	Standard deviation
TIA	Transient ischemic attack

## Appendix A. Standardized Clinical Scenarios

**Case 1**

**Chief Complaint:** “I have been having headaches for the past 6 months.”

**History of Present Illness:**

A 32-year-old female presents with a 6-month history of daily headaches. The headaches are described as throbbing, bilateral, and located in the temporal regions. The pain typically worsens with stress, physical exertion, and bright lights. The headaches are associated with nausea, vomiting, and photophobia. Over-the-counter pain relievers provide minimal relief. The patient denies any recent head trauma, fever, stiff neck, or focal neurological symptoms.

**Past Medical History:**

- Migraine headaches since adolescence, previously well-controlled with sumatriptan.
- Seasonal allergies.
- No history of asthma, diabetes, hypertension, or cardiac disease.

**Surgical History:**

- Tonsillectomy and adenoidectomy at age 5.

**Social History:**

- Smokes 1 pack of cigarettes per day for the past 10 years.
- Drinks alcohol socially on weekends.
- Denies illicit drug use.
- Employed as a teacher.
- Lives in an apartment with her husband and two children.

**Family History:**

- Mother has a history of migraine headaches.
- Father has a history of hypertension and hyperlipidemia.

**Review of Systems:**

- **General:** No weight loss or fever.
- **HEENT:** Headaches as described above. No visual disturbances, hearing loss, or tinnitus.
- **Cardiovascular:** No chest pain, palpitations, or shortness of breath.
- **Pulmonary:** No cough, sputum production, or dyspnea.
- **Gastrointestinal:** Occasional constipation.
- **Genitourinary:** No urinary frequency, urgency, or dysuria.
- **Musculoskeletal:** No joint pain, stiffness, or weakness.
- **Neurological:** Headaches as described above. No numbness, tingling, weakness, or difficulty speaking.
- **Psychiatric:** Occasional anxiety and insomnia.

**Physical Examination:**

- **General:** Well-developed, well-nourished female in no acute distress.
- **Vitals:** Temperature 37.0 °C, pulse 80 bpm, respirations 16 breaths/min, blood pressure 120/80 mmHg, oxygen saturation 99% on room air.
- **HEENT:** Pupils equal, round, reactive to light and accommodation. No fundoscopic abnormalities.
- **Neck:** Supple, no lymphadenopathy, no jugular venous distention.
- **Cardiovascular:** Regular rate and rhythm, no murmurs, rubs, or gallops.
- **Pulmonary:** Clear to auscultation bilaterally.
- **Abdomen:** Soft, non-tender, non-distended, no organomegaly.
- **Neurological:**
  ○ Cranial nerves II–XII intact.
  ○ Motor strength 5/5 bilaterally in all extremities.
  ○ Sensation intact to light touch, pinprick, and vibration.
  ○ Reflexes 2+ symmetrically.
  ○ Gait normal.
  ○ No cerebellar signs.

**Neurological Examination:**

- **Mental Status:** Alert and oriented x3.
- **Cranial Nerves:**
  ○ II: Visual acuity 20/20 in both eyes. Visual fields full to confrontation.
  ○ III, IV, VI: Extraocular movements intact.
  ○ V: Facial sensation intact to light touch and pinprick.
  ○ VII: Facial symmetry intact.
  ○ VIII: Hearing intact to whispered voice.
  ○ IX, X: Uvula midline, gag reflex present.
  ○ XI: Shoulder shrug and head turn strength equal bilaterally.
  ○ XII: Tongue midline, no fasciculations.
- **Motor:**
  ○ Upper and lower extremity strength 5/5 bilaterally.
  ○ No pronator drift.
- **Sensory:**
  ○ Light touch, pinprick, vibration, and proprioception intact in all extremities.
- **Reflexes:**
  ○ Biceps, triceps, brachioradialis, patellar, and Achilles reflexes 2+ bilaterally.
  ○ Plantar reflexes flexor bilaterally.
- **Coordination:**
  ○ Finger-to-nose, heel-to-shin, and rapid alternating movements normal.
  ○ Romberg test negative.
  ○ Gait normal.

**Assessment:**

- Chronic daily headaches, likely migraine.
- Possible medication overuse headache.
- Rule out other secondary causes of headache.

**Differential Diagnosis:**

- Migraine headaches.
- Medication overuse headache.
- Tension-type headaches.
- Cluster headaches.
- Sinus headaches.
- Subarachnoid hemorrhage.
- Brain tumor.
- Giant cell arteritis.

**Plan of Management:**

- Diagnostic Studies:
  ○ Complete blood count (CBC).
  ○ Comprehensive metabolic panel (CMP).
  ○ Erythrocyte sedimentation rate (ESR).
  ○ C-reactive protein (CRP).
  ○ Urinalysis.
  ○ Consider imaging studies (CT head or MRI head) if red flags are present.
- Treatment:
  ○ Medications:
    ♣ Discontinue any over-the-counter pain medications.
    ♣ Start prophylactic migraine medications (e.g., propranolol, topiramate).
    ♣ Prescribe acute migraine medications (e.g., sumatriptan, triptans).
  ○ Lifestyle modifications:
    ♣ Stress management techniques (e.g., relaxation, yoga).
    ♣ Regular sleep schedule.
    ♣ Avoidance of triggers (e.g., caffeine, alcohol, stress, bright lights).
    ♣ Regular exercise.
- Follow-up:
  ○ Schedule follow-up appointment in 1–2 weeks to assess headache response to treatment.

**Case 2**

This is a 68-year-old right-handed Caucasian female presenting to the clinic today with a chief complaint of worsening memory problems over the past year. The patient reports increasing difficulty remembering recent events, such as where she placed her keys, appointments, and the names of new acquaintances. She also describes episodes of forgetfulness, such as leaving the stove on or repeating herself during conversations. The patient denies any history of head trauma, seizures, or significant alcohol use.

**Past medical history** includes hypertension, hyperlipidemia, and hypothyroidism, all well-controlled with medications.

**Surgical history** includes a cholecystectomy in 2010 and a right knee replacement in 2018.

**Social history** reveals the patient is a retired school teacher, widowed for 5 years, and lives independently in her own home. She is socially active, enjoys gardening, and attends a weekly book club. She denies any tobacco use or illicit drug use.

**Family history** is significant for Alzheimer’s disease in her mother and a history of stroke in her father.

**Review of systems** is otherwise unremarkable.

**Physical examination** reveals a well-nourished, alert, and oriented to person, place, and time elderly female. Vital signs are within normal limits. Cardiovascular, respiratory, and abdominal examinations are unremarkable.

**Neurological examination** demonstrates normal cranial nerve function. Motor strength is 5/5 bilaterally in all extremities. Deep tendon reflexes are 2+ symmetrically. Sensory examination is intact.

**Assessment:** This patient presents with a concerning decline in cognitive function consistent with a possible dementia.

**Differential diagnosis** includes:

- Alzheimer’s disease.
- Vascular dementia.
- Frontotemporal dementia.
- Lewy body dementia.
- Depression.
- Medication side effects.
- Vitamin B12 deficiency.
- Hypothyroidism.

**Plan of management** includes:

- Complete blood count (CBC), comprehensive metabolic panel (CMP), thyroid function tests (TSH), vitamin B12 and folate levels, and lipid profile.
- Neuropsychological testing to further evaluate cognitive function.
- Brain imaging studies, such as MRI or CT scan of the head.
- Referral to a neurologist for further evaluation and management.

**Case 3**

This is a 68-year-old right-handed Caucasian male who presents to the Emergency Department complaining of sudden onset of left-sided weakness and difficulty speaking that began approximately 30 min prior to arrival. He describes the weakness as a numbness and tingling in his left arm and leg, which he noticed while getting out of bed. He also reports that his speech became slurred and he had difficulty finding the right words to express himself. These symptoms resolved completely within 15 min. He denies any associated headache, dizziness, vision changes, or loss of consciousness.

**Past Medical History** is significant for hypertension, hyperlipidemia, and type 2 diabetes mellitus. He underwent a coronary artery bypass graft in 2018.

**Social History** reveals he is a retired construction worker, former smoker (quit 10 years ago), and occasional social alcohol drinker. He lives independently with his wife.

**Family History** is significant for hypertension and coronary artery disease in his father.

**Review of Systems** is otherwise negative except as noted above.

**Physical Examination** reveals a well-nourished, alert, and oriented elderly male in no acute distress. Vital signs are stable with BP of 150/95. BMI 27. **Cardiac exam** is unremarkable. **Pulmonary exam** is clear to auscultation bilaterally. **Abdominal exam** is soft, non-tender, with no organomegaly. **Neurological exam** on arrival is **normal**.

**Assessment:**

- **Transient ischemic attack (TIA)**: This diagnosis is strongly suspected given the acute onset of focal neurological deficits (left-sided weakness, dysarthria) followed by complete resolution within 24 h.
- **Possible risk factors:** Hypertension, hyperlipidemia, diabetes, smoking history, and family history of cardiovascular disease.

**Differential Diagnoses:**

- **Stroke:** While complete resolution of symptoms suggests TIA, a small ischemic stroke cannot be entirely ruled out.
- **Migraine with aura:** This can present with transient neurological symptoms, but the typical features of headache and visual disturbances are absent in this case.
- **Hypoglycemia:** This can cause transient neurological symptoms, but the patient denies any history of diabetes-related hypoglycemia.

**Plan of Management:**

- Immediate evaluation:
  ○ **Non-contrast head CT:** To rule out intracranial hemorrhage.
  ○ **Electrocardiogram (ECG):** To assess for cardiac arrhythmias.
  ○ **Blood tests:** Complete blood count, comprehensive metabolic panel, lipid profile, and coagulation studies.
  ○ **Carotid ultrasound:** To assess for carotid artery stenosis.
  ○ **Echocardiogram:** To evaluate for cardiac sources of embolism.
- **Antiplatelet therapy:** Initiate aspirin or clopidogrel immediately to reduce the risk of subsequent stroke.
- **Risk factor modification:**
  ○ **Blood pressure control:** Optimize antihypertensive medications.
  ○ **Glycemic control:** Tightly control blood sugar levels.
  ○ **Lipid management:** Initiate or adjust statin therapy.
  ○ **Smoking cessation counseling:** If applicable.
  ○ **Dietary and lifestyle modifications:** Encourage a healthy diet and regular exercise.
- **Urgent neurology consultation: For further evaluation and management.**

**Case 4**

**Chief Complaint:** “My hands are shaking uncontrollably, and it is getting worse.”

**History of Present Illness:**

A 68-year-old right-handed Caucasian female presents with a 2-year history of progressively worsening tremors in both upper extremities. The tremors initially began subtly, with occasional hand shaking, but have gradually intensified. She describes them as resting tremors that worsen with stress and improve with purposeful movement. The tremors interfere with daily activities such as eating, drinking, writing, and buttoning clothes. She denies any associated weakness, numbness, tingling, speech difficulties, or gait instability.

**Past Medical History:**

- Hypertension, well-controlled on lisinopril.
- Hyperlipidemia, well-controlled on atorvastatin.
- Osteoarthritis of the knees.
- Hypothyroidism, well-controlled on levothyroxine.

**Surgical History:**

- Hysterectomy in 2005.
- Cholecystectomy in 2010.
- Cataract surgery bilaterally in 2018.

**Social History:**

- Retired school teacher.
- Lives independently in a single-family home.
- Denies smoking or illicit drug use.
- Occasional social alcohol consumption (1–2 glasses of wine per week).
- No significant travel history.

**Family History:**

- Mother: History of hypertension and hyperlipidemia.
- Father: History of Parkinson’s disease.
- Siblings: No significant medical history.

**Review of Systems:**

- **General:** No weight loss, fever, or fatigue.
- **HEENT:** No visual disturbances, hearing loss, tinnitus, epistaxis, sore throat, hoarseness, or dental problems.
- **Cardiovascular:** No chest pain, palpitations, shortness of breath, or edema.
- **Respiratory:** No cough, sputum production, or shortness of breath.
- **Gastrointestinal:** No abdominal pain, nausea, vomiting, diarrhea, or constipation.
- **Genitourinary:** No urinary incontinence, frequency, or urgency. No dysuria.
- **Musculoskeletal:** Occasional joint pain in knees, relieved with over-the-counter pain relievers.
- **Neurological:** Tremors in both upper extremities as described above. No headache, dizziness, vertigo, syncope, seizures, weakness, numbness, or tingling.
- **Psychiatric:** No anxiety, depression, or insomnia.

**Physical Examination:**

- **General:** Well-nourished, alert, and oriented x3.
- **Vital Signs:** Blood pressure 128/78 mmHg, pulse 72 beats/min regular, respirations 16 breaths/min, temperature 98.6 °F (37.0 °C), oxygen saturation 99% on room air.
- **Neurological Examination:**
  ○ **Mental Status:** Alert and oriented x3. Speech clear and fluent. No cognitive deficits.
  ○ **Cranial Nerves:** II-XII grossly intact.
  ○ **Motor:**
    ♣ **Strength:** 5/5 in all extremities.
    ♣ **Tone:** Normal.
    ♣ **Coordination:**
      - **Finger-to-nose:** Difficulty due to tremor.
      - **Rapid alternating movements:** Slow and clumsy.
      - **Heel-to-shin:** Intact.
    ♣ **Gait:** Normal.
    ♣ **Posture:** Upright.
    ♣ **Resting tremor:** Present in both hands at rest, pill-rolling in appearance.
    ♣ **Action tremor:** Minimal.
  ○ **Sensory:** Light touch, pinprick, vibration, and proprioception intact.
  ○ **Reflexes:** 2+ symmetrically in upper and lower extremities.
  ○ **Babinski sign:** Absent bilaterally.

**Assessment:**

- Tremor, likely Parkinson’s disease.

**Differential Diagnosis:**

- Essential tremor.
- Parkinson’s disease.
- Cerebellar tremor.
- Dystonia.
- Medication-induced tremor (e.g., beta-blockers, theophylline).
- Hyperthyroidism.
- Wilson’s disease.

**Plan of Management:**

1. **Diagnostic Workup:**
  - **Laboratory tests:** Complete blood count, comprehensive metabolic panel, thyroid function tests, liver function tests, and ceruloplasmin level.
  - **Neuroimaging:** Brain MRI with and without contrast.
  - **DaTscan:** To assess dopamine transporter function.
2. **Treatment:**
  - **Medical management:**
    - If confirmed Parkinson’s disease, initiate treatment with levodopa/carbidopa.
    - Consider beta-blockers (e.g., propranolol) for essential tremor.
  - **Non-pharmacological interventions:**
    - Occupational therapy for adaptive strategies to cope with tremors.
    - Physical therapy for exercise and balance training.
3. **Follow-up:**
  - Schedule a follow-up appointment in 1–2 weeks to discuss diagnostic results and further management options.

**Case 5**

This is a 58-year-old right-handed Caucasian male presenting to the clinic today with complaints of right lower back pain radiating down the right leg for the past three weeks. He describes the pain as a constant, dull ache with intermittent sharp, shooting pains that worsen with coughing, sneezing, and prolonged sitting. The pain extends from his low back down the posterior aspect of his right thigh, lateral aspect of his right calf, and into the fourth and fifth toes of his right foot. He denies any numbness, tingling, or weakness in his lower extremities. He reports difficulty sleeping due to the pain and has had to limit his usual activities, such as walking and golfing. He denies any recent trauma or falls.

Past medical history is significant for hypertension, hyperlipidemia, and type 2 diabetes mellitus. He underwent a right knee arthroscopy for meniscus repair five years ago. Surgical history is otherwise unremarkable. Social history reveals he is a retired school teacher, married, and lives in a single-story home. He denies smoking, illicit drug use, or excessive alcohol consumption. He maintains a healthy diet and exercises regularly with light walking and yoga. Family history is significant for hypertension and hyperlipidemia in both parents. Review of systems is otherwise unremarkable except for the above-mentioned back and leg pain.

On physical examination, vital signs are within normal limits. Inspection of the back reveals no signs of deformity or erythema. Palpation of the lumbosacral spine reveals tenderness to palpation over the L5-S1 region. Range of motion of the lumbar spine is limited in all directions with associated pain. Straight leg raise test is positive on the right at 30 degrees with radiation of pain down the right leg. Neurological examination reveals normal motor strength in all extremities. Deep tendon reflexes are 2+ bilaterally in the upper and lower extremities. Sensation to light touch and pinprick is intact in all extremities. Babinski’s sign is absent bilaterally. Gait is normal.

Assessment: The patient presents with a clinical picture consistent with right L5-S1 radiculopathy.

Differential Diagnosis: Other potential diagnoses include lumbar spinal stenosis, piriformis syndrome, and diabetic peripheral neuropathy.

Plan of Management:

- Conservative management:
  ○ Pain management: Over-the-counter pain relievers such as acetaminophen or ibuprofen. Consider referral to physical therapy for modalities such as heat therapy, massage, and core strengthening exercises.
  ○ Lifestyle modifications: Encourage weight loss if overweight or obese. Advise the patient to maintain good posture and avoid prolonged sitting or standing.
- Imaging studies: Obtain lumbar spine MRI to confirm the diagnosis and rule out other potential causes.
- Further evaluation: If conservative management fails to improve symptoms, consider further evaluation with a neurologist or neurosurgeon.

## Appendix B. Sample AI-Generated Neurology Referral Letters

### Appendix B.1. High-Quality Referral Letter (TIA Case–Letter 3.10, Score: 30/30)

**[Referring Physician’s Name]**

Family Medicine Physician

[Clinic/Hospital Name]

[Clinic Address]

[City, State, ZIP Code]

[Date]

**Neurology Consultant**

[Neurology Clinic Name]

[Neurologist’s Address]

[City, State, ZIP Code]

**Re: Neurology Referral for Transient Ischemic Attack Evaluation**

**Patient Name:** [Patient’s Full Name]

**Date of Birth:** [Patient’s DOB]

Dear Dr. [Neurologist’s Last Name],

I am referring Mr. [Patient’s Last Name], a 68-year-old right-handed male, for evaluation of a suspected **transient ischemic attack (TIA)** that occurred earlier today. Below is a summary of his presentation and findings:

**History of Present Illness**

Mr. [Last Name] experienced sudden-onset **left-sided weakness and difficulty speaking** approximately **30 min** before arrival at my clinic. He described numbness and tingling in his left arm and leg, as well as **slurred speech and word-finding difficulty**. These symptoms **fully resolved within 15 min**. He denies headache, dizziness, vision changes, or loss of consciousness.

His past medical history includes **hypertension, hyperlipidemia, and type 2 diabetes mellitus**. He underwent **coronary artery bypass grafting in 2018**. He is a **retired construction worker**, a **former smoker (quit 10 years ago),** and an **occasional social alcohol drinker**.

**Physical Examination**

- **General:** Well-nourished, alert, and oriented male in no acute distress.
- **Vital Signs:** BP 150/95 mmHg, HR 78 bpm, RR 16, BMI 27.
- **Cardiac & Pulmonary:** Regular rate and rhythm; clear to auscultation.
- **Neurological Exam: Normal on arrival**; no motor or sensory deficits.

**Initial Workup & Differential Diagnosis**

**Primary Diagnosis:** Suspected TIA. Given his risk factors (hypertension, diabetes, hyperlipidemia, smoking history), his symptoms warrant further evaluation.

**Differential Diagnoses:**

- Stroke (though complete symptom resolution suggests TIA).
- Migraine aura without headache.
- Hypoglycemia (unlikely given normal blood glucose).

**Plan & Referral Request**

I am requesting **urgent neurology consultation** to further assess stroke risk and management. Workup and next steps include:

- Immediate imaging: Non-contrast head CT to rule out hemorrhage.
- Vascular studies: Carotid ultrasound for stenosis evaluation.
- Cardiac assessment: Echocardiogram, ECG to assess embolic sources.
- Risk factor management: Initiation of aspirin/clopidogrel, blood pressure control.

Your expertise in determining **long-term secondary prevention strategies** and further workup (e.g., MRI brain, MRA) would be highly valuable. Please let me know if additional information is needed.

Thank you for your evaluation and guidance.

**Sincerely,**

[Your Full Name, MD]

[Your Contact Information]

### Appendix B.2. Low-Quality Referral Letter (Radiculopathy Case–Letter 5.2, Score: 10/30)

**[Referring Physician’s Name]**

Family Medicine Physician

[Clinic/Hospital Name]

[Clinic Address]

[City, State, ZIP Code]

[Date]

**Neurology Consultant**

[Neurology Clinic Name]

[Neurologist’s Address]

[City, State, ZIP Code]

**Re: Neurology Consultation for [Patient’s Name]**

Dear Dr. [Neurologist’s Last Name],

I am referring **[Patient’s Name], a [Age]-year-old [Gender]**, for further neurological evaluation. He presents with **[brief summary of symptoms]**, which have persisted for **[duration]**.

**Clinical History**

- **Presenting Complaint: [Details of symptoms, onset, progression, severity, any related factors]**
- **Past Medical History: [Relevant conditions, surgeries, or major illnesses]**
- **Medications: [Current medications, dosages, recent changes]**
- **Family History: [Pertinent neurological or other conditions]**

**Physical Examination**

- General Appearance: [Patient’s general condition, distress, etc.]
- Neurological Examination: [Summary of findings: mental status, cranial nerves, motor function, reflexes, gait]

**Investigations & Consultation Request**

**[List any prior workup performed, e.g., imaging, labs, pending tests]**

I am requesting **[neurologist’s input regarding differential diagnoses, further workup, and management options]**.

Please let me know if you need any further details. Thank you for your assessment and recommendations.

**Sincerely,**

[Your Full Name, MD]

[Your Contact Information]

**Commentary for Appendix B**

- Letter 3.10 demonstrates strong organization, completeness, and clinical clarity, scoring 30/30.
- Letter 5.2 lacks key details in history, physical examination, and plan, as the LLM left the information areas incomplete (10/30).

These examples highlight the variability in AI-generated referral letters and emphasize the importance of physician oversight in clinical documentation.
